# Low Dielectric Constant Photocurable Fluorinated Poly (Phthalazinone Ether) Ink with Excellent Mechanical Properties and Heat Resistance

**DOI:** 10.3390/polym15061531

**Published:** 2023-03-20

**Authors:** Guangsheng Zhang, Chenghao Wang, Lingmei Jiang, Yibo Wang, Bing Wang, Xiaoxu Wang, Haoran Liu, Lishuai Zong, Jinyan Wang, Xigao Jian

**Affiliations:** 1 School of Chemical Engineering, Dalian University of Technology, Dalian 116024, China; 2 State Key Laboratory of Fine Chemicals, Dalian University of Technology, Dalian 116024, China; 3 Technology Innovation Center of High Performance Resin Materials, Dalian 116024, China; 4 School of Materials Science and Engineering, Dalian University of Technology, Dalian 116024, China

**Keywords:** photosensitive resin, mechanical properties, heat resistance, dielectric constant

## Abstract

The photosensitive resins for 3D printing technology have been widely applied throughout the advanced communication field due to their merits of high molding accuracy and fast processing speed. Regardless, they, in particular, should have better mechanical properties, heat resistance, and dielectric properties. Herein, photocurable fluorinated poly (phthalazinone ether) (FSt-FPPE) was utilized as a prepolymer to improve the performance of photosensitive resin. A series of UV-curable inks named FST/DPGs were prepared with FSt-FPPE and acrylic diluents of different mass fractions. The FST/DPGs were cured into films by UV curing and post-treatment. After curing, their properties were characterized in detail. In terms of heat resistance, glass transition temperature (*T_g_*) could reach 233 °C and the 5% thermal decomposition temperature (*T_d5%_*) was 371 °C. The tensile strength surprisingly reached 61.5 MPa, and the dielectric constant (*D_k_*) could be significantly reduced to 2.75. Additionally, FST/DPGs were successfully employed in UV-assisted direct writing (DIW) to print 3D objects that benefited from their commendable fluidity and rapid curing speed. A stiff cylinder sample with a smooth surface and distinct pattern was ultimately obtained, indicating their remarkable 3D printing adaptation. Such photosensitive resin for UV-assisted DIW exhibits tremendous potential in the electronic industry.

## 1. Introduction

With the rapid development of 5G communication networks, the high-speed communication field sets higher requirements for applying low-dielectric materials [1,2,3,4]. Low dielectric materials as the substrate in the 5G era can ensure that the signal in the transmission process is able to maintain high speed and high fidelity. Organic polymer materials (such as polyimide [5], polyaryl ether, and cyanate ester) have great potential in the field because of their low *D_k_* and superior mechanical properties [6,7,8,9]. Their accuracy is difficult to achieve through traditional processing methods, such as subtractive manufacturing (SM). Three-dimensional (3D) printing, also known as additive manufacturing (AM), is a speedy manufacturing technology that can fundamentally subvert the traditional method of converting materials into complex equipment efficiently and flexibly [10,11,12]. As the most advanced 3D printing technology, UV curing 3D printing, including direct writing (DIW), stereolithography (SLA), and the PolyJet additive technology [13] constructs 3D objects by UV laser photocuring liquid photosensitive resin layer by layer. However, the inherent brittleness, poor heat resistance, and dielectric properties of photosensitive resin limit its development in the communication field [14,15,16]. Therefore, enhancing its mechanical and thermal properties is critical.

Suffering from the weaknesses of poor heat resistance and mechanical properties of photosensitive resin, researchers recently used a variety of enhancement techniques [17,18]. Liu et al. [19] formed an interpenetrating polymer network (IPNs) of isocyanate/polyol by UV-hot double-curing, and the maximum tensile strength of the material was 17.5 MPa. Guo et al. [20] designed maleic anhydride-terminated polyimide oligomers, and the tensile strength was 25 MPa after UV curing. Wei et al. [21] synthesized isosorbitol di(methyl) acrylate (ISD(M)A) as a photocuring active diluent through molecular design. Compared with the aliphatic chain diluted monomer, the *T_g_* and tensile strength of the system could be increased by 15~20 °C and 10 MPa when ISDA was used as the diluted monomer. According to previous studies, the mechanical properties and heat resistance of the photosensitive resin have a narrow advancement through different designs. However, the application in the communication field is not sufficient, and the dielectric properties of the photosensitive resin are rarely reported.

Fluorinated poly(phthalazinone ether) s (FPPEs) [22], as an engineering plastic, had the advantages of high temperature resistance, high strength, low dielectric constant, and good solubility. Therefore, FPPE was capped with pentafluorostyrene to obtain FST-FPPE, which was used as the prepolymer of the photosensitive resin system. Dipropylene glycol diacrylate (DPGDA) was used as the dilution monomer, 1-hydroxycyclohexyl phenyl ketone (HCPK) acted as the photoinitiator, and triethanolamine (TEOA) was applied as the antioxygen. They were dissolved with FSt-FPPE in the mixed solution of acetone and cyclohexanone to obtain photosensitive resin. A series of curing films was obtained under UV light irradiation, and then the properties of the curing films were characterized. The introduction of FST-FPPE, which could introduce rigid structures into the system through establishing a high strength and toughness cross-linked network [23,24] with DPGDA, greatly enhanced the photosensitive resin’s mechanical properties and thermal properties. It was proven that it had a broad application prospect in the electronic material dielectric layer field, such as circuit boards.

## 2. Experimental Section

### 2.1. Materials

The materials used in this article are shown in Table 1.

### 2.2. Eexperimental Methodology

#### 2.2.1. Synthesis of FSt-FPPE

Using DHPZ and HFBPA as bisphenol monomer, DFBP as bishalogen monomer, and PFST as a capping agent, FSt-FPPE with a molecular weight of 6000 g/mol was synthesized by a solution polycondensation reaction. The synthetic route of the prepolymer is shown in Figure S1.

DHPZ (2.7244 g, 11.4 mmol), HFBPA (2.2831 g, 6.8 mmol), and K2CO3 (3.5264 g, 25.5 mmol) were mixed well. Then, 25 mL toluene was added. Afterwards, the water was removed through the addition of toluene at 130–135 °C for 3 h, which brought the reaction down to 110 °C, followed by the addition of PFST (345 μL, 2.5 mmol) and DMAc (10 mL) for 1 h, and finally increased to 130 °C, followed by the addition of DFBP (5.6718 g, 17 mmol). After 10 h, the viscosity of the system didn’t rise again. The reaction solution was immersed in boiling water containing a small amount of hydrochloric acid, resulting in a white granular polymer, repeatedly boiled and dried, dissolved in chloroform. The white powder polymer was immersed in ethanol and dried at 120 °C for 24 h in a vacuum oven. The yield was 85%.

#### 2.2.2. Purification of DPGDA

DPGDA used in this experiment contained a polymerization inhibitor (phenol), so the polymerization inhibitor in the monomer should be removed first when the photosensitive resin was configured; otherwise, the UV curing activity would be affected. The removal was carried out in an alkaline alumina chromatographic column.

To activate the alkaline alumina, 20 g of alkaline alumina were dried for 5 h in a 250 °C oven, then the dried alumina was weighed, 4% (mass fraction) of deionized water was added, and the mixture was stirred evenly, ground, crushed, and stored for 24 h.

To purify the monomer, the purchased DPGDA was poured into a chromatographic column containing activated alkaline alumina, and the dripping liquid was filtered to obtain pure DPGDA.

#### 2.2.3. Preparation of Photosensitive Resin

The FSt-FPPE was added to the mixed solvent of acetone and cyclohexanone, with a volume ratio of acetone to cyclohexanone of 1:2.5, to form a solution with a solid content of 0.4545%. Then, a different content of DPGDA was added and mixed evenly. Finally, HCPK and TEOA, which accounted for 3% and 4% of the total mass of FSt-FPPE and DPGDA, respectively, were added to the resin and thoroughly stirred to obtain a series of UV-curable inks named FST/DPGs. The results are shown in Table 2.

#### 2.2.4. UV Curing and Postprocessing

Photosensitive resins were cast on glass plates. All the formulations were UV-cured for 40 s at an intensity of 34 mW·cm^−2^ with a UV oven (XLite 600F), at a distance of 20 cm away from the mercury lamp.

The UV-cured objects were placed in a vacuum oven to remove the solvent in the films after UV curing and treated with the following curing process: 80 °C/2 h, 100 °C/2 h, 150 °C/8 h. Finally, the objects were cooled naturally to room temperature.

#### 2.2.5. Printing Parameters and Post-Processing of 3D Sample

**Figure 1:**
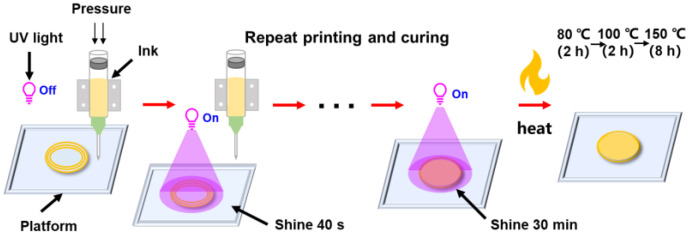
Schematic illustration of the 3D printing photosensitive resin.

The 3D printing machine model was a JD200 Jet with a nozzle diameter of 0.31 mm and a print height of 0.15 mm per layer through DIW. The 3D printing process was shown in Figure 1. The printing speed of the samples was 20 mm/s at a stable 0.2 MPa air pressure. Under the UV-curing machine, with a frequency of 365 nm and power of 36 W, the photosensitive resin was cured for 40 s after each layer was printed, and when the total printing height was 3 mm, the printing was stopped, followed by UV irradiation for 30 min to cure completely. Then they were placed in a vacuum oven for 80 °C/2 h, 100 °C/2 h, and 150 °C/8 h after UV curing. Finally, the samples were cooled naturally to room temperature.

### 2.3. Characterizations

All 1H-NMR spectra were recorded on a Bruker Vaian DLG400 spectrometer. Using tetramethyl silane (TMS) as an internal standard, 5–10 mg of each compound were dissolved in 0.5 mL of deuterochloroform solution at room temperature. Fourier transform infrared (FTIR) measurements were performed using a Thermo Nicolet IS50 FTIR spectrometer. The dynamic viscosity of the photosensitive resin was measured at 25 °C with an SNB-1 rotational viscometer. Thermogravimetric analysis (TGA) was performed using a Mettler thermogravimetric analyzer (Mettler TGA) at a heating rate of 20 °C/min in nitrogen. A Mettler DMA/SDTA 8610e measuring instrument was used to measure the film thickness of 9 mm × 3 mm × 0.05 mm; the frequency was 1 Hz; the heating rate was 3 °C/min; and the range was 30–250 °C. The mechanical properties of the 20 mm × 6 mm × 0.05 mm film samples were tested using an Instron5942 universal testing machine with a 50 N transducer and a tensile rate of 5 mm/min. Wide-angle X-ray diffraction (WAXD) was measured with a Smart Lab 9 kW diffractometer at room temperature with 10°/min rotation angular rate, Cu Kα radiation (240 kV, 50 mA). The tensile fracture of FST/DPGs films was investigated by a field-emission scanning electron microscope system (Hitachi, SU8220, Tokyo, Japan). The dielectric constant (*D_k_*) and loss (*D_f_*) were measured with a Keysight 5080B network analyzer at 25 °C at a frequency of 20 GHz. The angle of contact of the films with water was measured with a contact angle measuring instrument (JCD2000D2W), which measured from 0° to 180°, and the measurements were performed with 5 μL drops of water on the surface of flat-cured film at room temperature.

## 3. Results

### 3.1. FTIRanalysis and Gel Content Analysis of FST/DPGs

**Figure 2:**
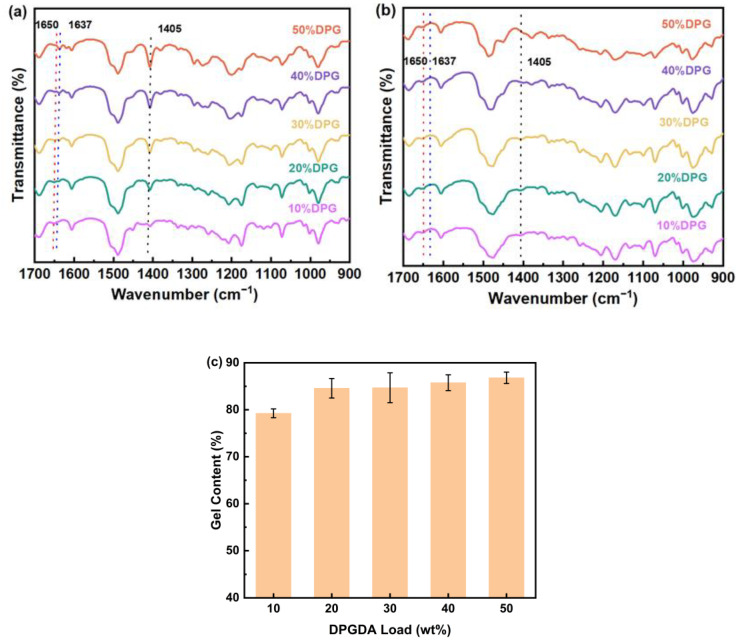
FTIR spectrum of (**a**) uncured FST/DPGs and (**b**) cured FST/DPGs; (**c**) gel content of the FST/DPGs cured films.

The degree of UV curing reaction was analyzed qualitatively using FTIR. The FTIR spectra of uncured FST/DPGs and cured FST/DPGs are shown in Figure 2a,b. In the spectrum of uncured FST/DPGs, the peak at 1687 cm^−1^ was attributed to the stretching vibration of the carbonyl group in FST-FPPE, which increased as the DPGDA ratio decreased. The peak at 1650 cm^−1^ was ascribed to C = N in FST-FPPE, and the peaks at 1637 cm^−1^ and 1405 cm^−1^ indicated C = C bonds in FST-FPPE and DPGDA [25]. In the FTIR spectra of the cured FST/DPGs, it was found that the peaks at 1637 cm^−1^ and 1405 cm^−1^ disappeared after UV curing. By this phenomenon, it could be assumed that the reaction of the photosensitive resin under UV curing was complete.

The degree of the UV curing reaction was quantified by measuring the gel content of the cured films. The cross-linked part of the cured films was reported to be insoluble in chloroform, and the rest was soluble. In this study, the gel content of the cured films was calculated by weighing them. The specific method was to weigh each UV-cured film, after it had been soaked in a Soxhlet extractor filled with chloroform for 12 h, and then completely dried in a blast furnace at 120 °C before being weighed [26,27]. Gel content (G) was calculated using Equation (1):

(1) $$G=\frac{w_{1}}{w_{0}}\times 100\%$$

where *w*_0_ is the weight before chloroform soaking (g), and w_1_ is the weight after soaking and drying (g). Figure 2c shows the gel content of the cured films. It was observed that the gel content increased from 79.2 to 86.8% as the samples went from 10% DPG to 50% DPG. When the content of DPGDA in the system was low, the high rigidity of FST-FPPE limited the chain movement, resulting in lower polymerization and gel content [26]. On the contrary, not only was the curing activity of the whole system greatly enhanced, but the flexibility of the system was also improved, which resulted in a higher gel content of the system. Thus, the quality of the dilution monomer added to the system influenced the degree of curing of the system.

### 3.2. Mechanical Properties of FST/DPGs

**Figure 3:**
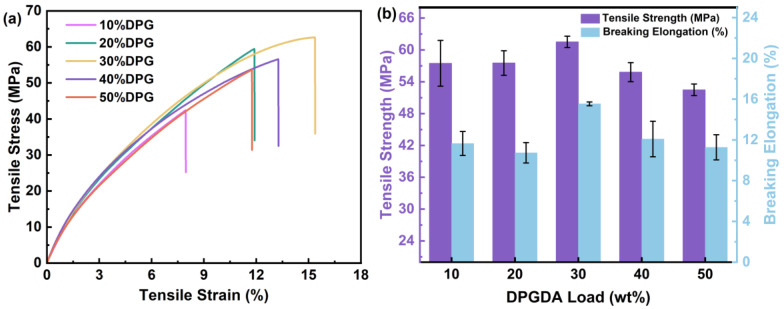
(**a**) Stress–strain curves; (**b**) tensile strength and breaking elongation of the FST/DPGs cured films.

The effects of different compositions of diluted monomer and prepolymer on the mechanical properties of the photosensitive resin were investigated by tensile tests, and the results are shown in Figure 3. From Figure 3b, it can be seen that the highest tensile strength and elongation at break were 61.5 MPa and 15.5%, respectively, when the amount of DPGDA was 30 wt.%. The movement between molecular chains in the cured network was restricted to increase the tensile strength, while the long, rigid chain structure of FSt-FPPE had a large free volume, which facilitated the increase in elongation at break [28]. However, when the amount of DPGDA was higher than 30 wt.%, the tensile strength and elongation at break decreased. As short aliphatic chains, the high content of DPGDA not only destroyed the rigidity of the system, but also severely restricted the movement between molecular chains as the cross-link density increased, all of which deteriorated the mechanical properties of the films. [29]. These results indicated that the addition of FST-FPPE to the photosensitive resin system could significantly change the tensile strength and elongation at the break of the cured films.

### 3.3. SEM Analysis of FST/DPGs

**Figure 4:**
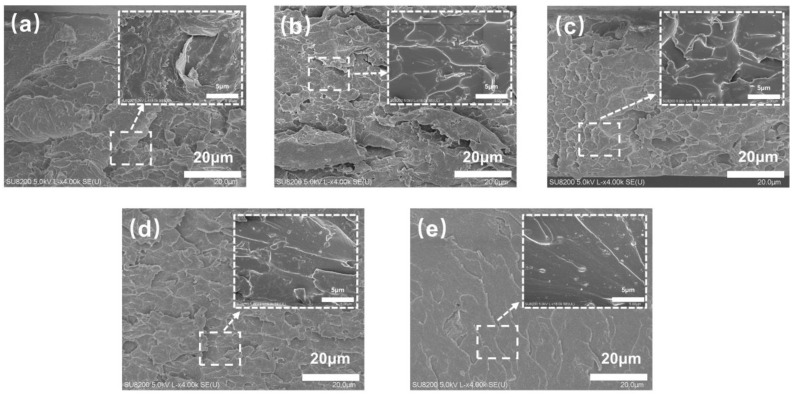
SEM images of the FST/DPGs-cured films. (**a**) 10% DPG; (**b**) 20% DPG; (**c**) 30% DPG; (**d**) 40% DPG; (**e**) 50% DPG.

The tensile fracture morphology of FST/DPGs was observed using SEM, as shown in Figure 4. When the samples were from 10% DPG to 30% DPG (Figure 4a–c), the cross section of the films became rough, and many large and small gullies appeared incidental to the direction of crack expansion, which allowed more energy to be absorbed during the formation and development of a large number of cracks and gullies under tensile conditions, thus leading to the enhancement of the toughness and strength of the materials [30,31,32]. This result was closely related to the increase in DPGDA content, which enhanced the crosslink density of the system, with enough segments in the system to act as a toughening agent and improve the tensile strength of the cured films. The cracks generated by stretching gradually decreased when the samples were changed from 30% to 50% (Figure 4c–e). When the cured film was 50% DPG, the number of cracks was the least, and the direction of the cracks was single, resulting in no dispersed stress between cracks. The fracture mode of the films gradually changed from ductile to brittle. This was due to the high cross-link density of the films due to the high content of DPGDA, which severely restricted the movement of the molecular chains, and the decrease in the prepolymer content, which led to the deterioration of the toughening effect of the system. Based on the above analysis, the pattern of the mechanical property data of the cured films was also proven.

### 3.4. Thermal Characterization of FST/DPGs

**Figure 5:**
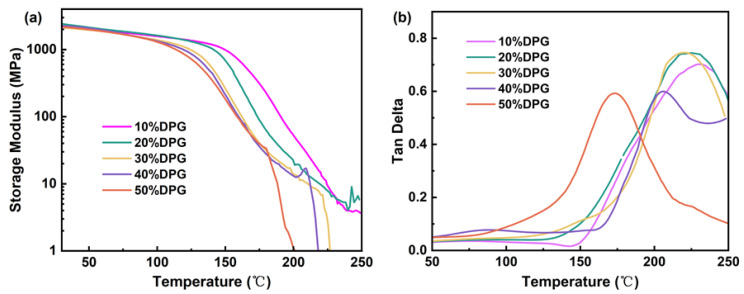
(**a**) DMA curve of the FST/DPGs cured films, and (**b**) tan δ curves of the FST/DPGs cured films.

The dynamic thermomechanical properties of the materials were evaluated using DMA. Figure 5 shows the tan δ and storage modulus curves of the cured films. These data are shown in Table 3. The temperature corresponding to the tan δ peak was *T_g_* of the film [33]. It can be seen from Figure 5b that the cured films had excellent heat resistance. As the content of DPGDA in the system increased, the *T_g_* of the cured films decreased. The reason was that, since DPGDA was a flexible fat chain, the increase in its content would reduce the rigidity of the system, resulting in a decrease in the *T_g_* of the cured films. Ten percent DPG had the highest *T_g_*, which was 233 °C. These results indicate that the addition of FST-FPPE could greatly improve the heat resistance of the system.

**Figure 6:**
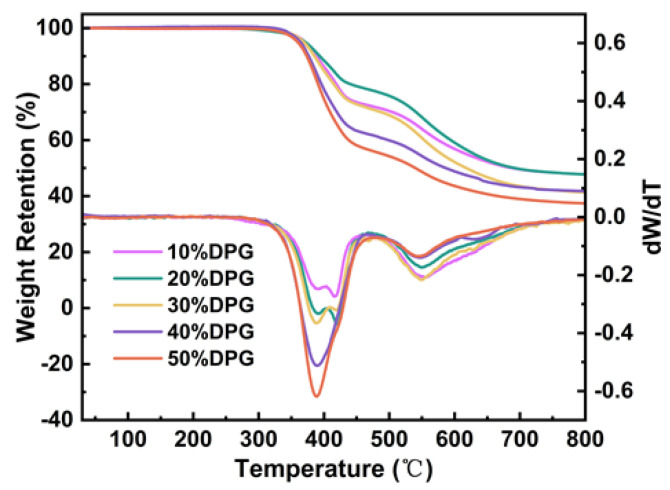
TGA and DTG curves of the FST/DPGs-cured films.

The thermal stability of the entirely cured films was evaluated by TGA. Figure 6 shows a graph of the weight loss of the cured films as a function of temperature and its derivative. As can be observed from Figure 6, for DPGDA content between 10 and 30%, there were three evident decomposition stages, and from 40 to 50%, there were only two decomposition stages, since, when the dilution monomer content was less than 30%, the cross-linking network in the system was uneven, generating different ester group decomposition temperatures in the acrylate unit. When the dilution monomer content was higher than 40%, the crosslinking network of the system was uniform, so the decomposition temperature of the ester group was the same, which was 389 °C. At the same time, there was a decomposition peak between 545 and 557 °C for different cured films, which was the decomposition of rigid structures, such as benzene rings, in the system. When the content of DPGDA was 10%, the highest 5% thermal decomposition temperature (*T_d5%_*) was 371 °C, the highest peak temperature of the fastest decomposition rate was 557 °C, and the highest carbon residue content was 48%. The rigid structure in the system improved the heat resistance of the cured films.

### 3.5. Dielectric Properties of FST/DPGs

**Figure 7:**
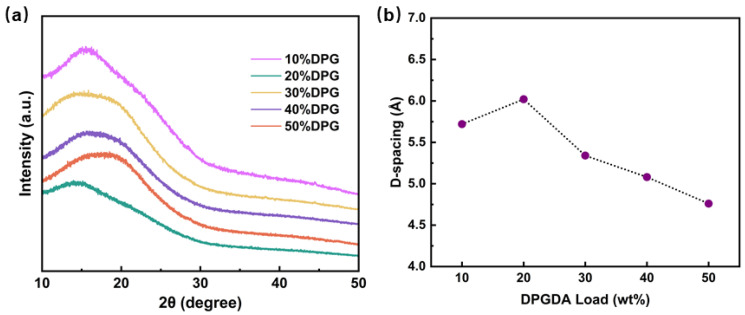
(**a**) The curves of WAXD characterization and (**b**) d-spacing of the FST/DPGs.

For dielectric materials in the field of high-speed communication, low *D_k_* could improve signal transmission speed and reduce the signal transmission loss. Table 4 shows the measured *D_k_* and *D_f_* of FST/DPGs-cured films @20GHz. Their *D_k_* was 2.75–2.95, and the *D_k_* and *D_f_* of the cured film with 20% DPG were the lowest, at 2.75 and 0.0193, respectively. This showed that the cured films had excellent dielectric properties at high frequencies. According to the previous research, the introduction of a cross-linked structure increased the free volume between molecular chains in the system, and the Clausius–Mosotti equation (Equation (2)) explains that the *D_k_* of the material decreased with the increase of its free volume, which could be represented by calculating its average interchain distance (d-spacing) [34]. Therefore, we performed WAXD characterization on the FST/DPGs-cured films. From Figure 7, it can be seen that the maximum 2θ values of the broad peaks of the FST/DPGs were shifted first to the left and then to the right. The d-spacing values were calculated by Bragg’s law (Equation (3)) based on the maximum 2θ values of the broad peaks. These results are summarized in Table 5 [35,36]. This demonstrated that the introduction of the cross-linked network increased the free volume of the photosensitive resin, reducing the *D_k_* when the amount of DPGDA was increased from 10 to 20%. With the increasing addition of diluent monomer, the crosslinking density of the system increased, but the free volume decreased. It originated from that high content led to crosslinking of the diluent monomer itself, which would fill into the crosslinked network, making the free volume of the system smaller resulting growing the *D_k_* when the amount of DPGDA was increased from 20 to 50%. The above results confirmed that the *D_k_* and *D_f_* of the system could be reduced by introducing an appropriate cross-linking network.

(2) $$D_{k}=\frac{1+2\left(\frac{P_{m}}{V_{m}}\right)}{1-\left(\frac{P_{m}}{V_{m}}\right)}$$

where *D_k_* is the dielectric constant, *P_m_* is the molar polarization of the molecular group (cm^3^·mol^−1^), and *V_m_* is the molar volume of the molecular group (cm^3^·mol^−1^), and

(3) d=λ/2sinθ

where d is the crystal plane spacing (nm), θ is the angle between the incident X-ray and the corresponding crystal plane (°), and λ is the wavelength of the X-ray (nm).

### 3.6. Water Contact Angle and Water Absorption of FST/DPGs

**Figure 8:**
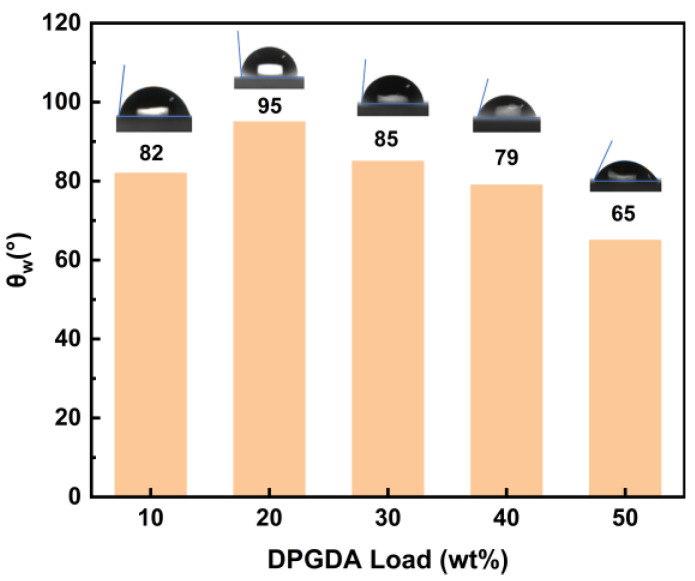
Contact angle of the FST/DPG with water.

For contact angles below 90°, the material is considered hydrophilic. Contact angles greater than 90° are defined as hydrophobic [37]. As shown in Figure 8, cured film with 20 wt.% DPGDA exhibited certain hydrophobic property. Both higher fluorine element and degree of cross-linking led to such high water contact angle. For the 20 wt.% or more DPGDA loading, all cured films possessed a smaller contact angle, stemming from the increasing C = O polar group contents and decreasing fluorine ratios. Table 6 illustrates the water absorption of the cured FST/DPGs. The 20 wt.% DPGDA sample showed the weakest water absorption capacity, originating from the amount of polar group and the density of crosslinking. The above results show that to reduce the water absorption, the hydrophobicity of the material could be effectively increased by reducing the polar group content and introducing cross-linked networks.

### 3.7. D Printing Sample

**Figure 9:**
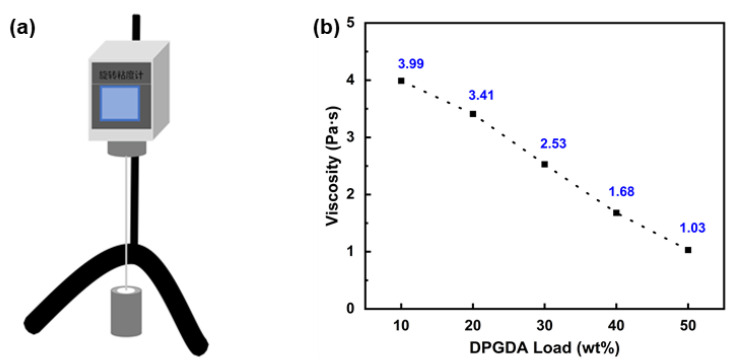
(**a**) Schematic diagram of rotational viscometer; (**b**) viscosity curve of the FST/DPGs.

**Figure 10:**
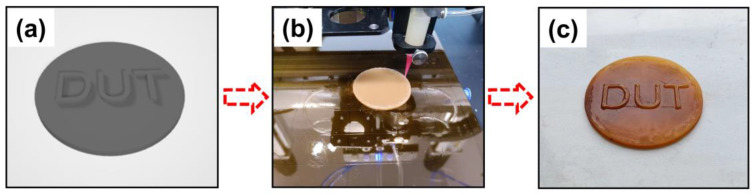
(**a**) 3D-printed model of the sample; (**b**) figure of the direct-writing process; (**c**) 3D-printed sample after UV curing and postprocessing.

To facilitate DIW 3D printing, the viscosity of FSTs/DPGs was measured using a rotational viscometer, as shown in Figure 9. The viscosity of the ink ranged from 1.03 to 3.99 Pa·s and decreased gradually with the increase in dilution monomer content. The flexible fatty chain structure of the dilution monomer was excellent at reducing the system’s viscosity. The 30% DPG was selected as the printing ink. As shown in Figure 10a, a 3D pattern was designed using C4D software, and the sample shown in Figure 10c was obtained by the printing process shown in Figure 10b after light curing and post-processing. The surface morphology of the model indicated that it ensured sufficient shape integrity and a relatively flat surface.

## 4. Conclusions

Pentafluorostyrene was introduced into fluorinated poly (phthalazinone ether) to endow it with photocurable ability, and a series of low-dielectric ink FST/DPGs was compounded with diluent and other components. After ultraviolet and post-treatment, cured films with excellent mechanical properties, heat resistance, and dielectric properties were obtained. When the content of DPGDA increased from 10 to 50 wt.%, the tensile strength increased first for the greater cross-linkage and then decreased due to the more flexible chain, up to 61.5 MPa (30% DPG), and the *T_g_* dropped from 233 °C to 174 °C owing to the structure having fewer phthalazinone moieties. The results showed that the introduction of FSt-FPPE could improve the strength and heat resistance of the system by forming a rigid cross-linking network structure. According to dielectric test, the dielectric constant of 20% DPG film was 2.75 @20 GHz for its low polarity units, large free volume, and poor polymer chain mobility derived from high crosslink density. In addition, the photosensitive resin, due to its lower viscosity and fast curing speed, is suitable for UV-assisted DIW. This study presents an innovative method for improving the mechanical properties and heat resistance of photosensitive resin, which has great potential in high-speed communication.

## Figures and Tables

**Table 1:** Main data and sources of the materials.

Materials	Abbreviations	Purity	Company
4-(4-hydroxyl-phenyl) (2H)-phthalazinone-1-one	DHPZ	>98%	Dalian Polymer New Material Co. Ltd.
4,4′-(Hexafluoroisopropylidene)diphenol	HFBPA	>98%	Shanghai Aladdin.
Pentafluorostyrene	PFST	>98%	Shanghai Aladdin.
Decafluorobiphenyl	DFBP	99%	Shanghai Macklin Inc.
Dipropylene glycol diacrylate	DPGDA	≥80%	Shanghai Macklin Inc.
Triethanolamine	TEOA	≥99%	Shanghai Aladdin.
1-Hydroxycyclohexyl phenyl ketone	HCPK	>95%	Shanghai Bide Pharmatech Ltd.
Acetone	-	-	Tianjin third chemical agent Co.
Cyclohexanone	-	-	Tianjin third chemical agent Co.
Dimethylacetamide	DMAc	-	Tianjin third chemical agent Co.

**Table 2:** Formulation of the UV-curable ink.

Samples	FSt-FPPE (wt.%)	DPGDA (wt.%)
10% DPG	90	10
20% DPG	80	20
30% DPG	70	30
40% DPG	60	40
50% DPG	50	50

**Table 3:** *T_g_* and main thermogravimetric data of the FST/DPGs cured films.

Samples	*E’* (MPa)	*T_g_* (°C)	*T_d_*_5%_ (°C)	*T_dmax_* (°C)	*Cy* (%)
10% DPG	2163	233	371	416	48
20% DPG	2412	225	371	420	48
30% DPG	2106	220	369	389	42
40% DPG	2262	206	364	389	42
50% DPG	2168	174	360	389	37

**Table 4:** Dielectric properties of the FST/DPGs (@20 GHz).

Samples	*D_k_*	*D_f_*
10% DPG	2.81	0.0212
20% DPG	2.75	0.0193
30% DPG	2.86	0.0234
40% DPG	2.95	0.0383
50% DPG	2.96	0.0418

**Table 5:** The results of WAXD characterization of the FST/DPGs.

	10% DPG	20% DPG	30% DPG	40% DPG	50% DPG
*2θ* (°)	15.48	14.69	16.58	17.43	18.61
*d-spacing* (Å)	5.72	6.02	5.34	5.08	4.76

**Table 6:** Water absorption of the FST/DPGs-cured films.

Samples	Weights ^1^ (g)	Weights ^2^ (g)	Water Absorption (%)
10% DPG	0.2731	0.2741	0.3662
20% DPG	0.3655	0.3663	0.2189
30% DPG	0.3769	0.3790	0.5572
40% DPG	0.4291	0.4330	0.9089
50% DPG	0.4089	0.4127	0.9293

^1^ After drying at 110 °C for 3 h. ^2^ After soaking at 30 °C for 24 h.

## Data Availability

Not applicable.

## Supplementary Materials

The following supporting information can be downloaded at: https://www.mdpi.com/article/10.3390/polym15061531/s1, Figure S1: The synthesis of FSt-FPPE; Table S1: Characterization of molecular weights of FSt-FPPE; Figure S2: 1H NMR spectra of FSt-FPPE; Figure S3: TGA and DSC curves of FSt-FPPE; Figure S4: The molecular structure for DPGDA, HPCK, and TEOA. Figure S5: Printing process and the image of the printed samples. Video S1: DIW process.

## References

[1] Evans A.M., Giri A., Sangwan V.K., Xun S., Bartnof M., Torres-Castanedo C.G., Balch H.B., Rahn M.S., Bradshaw N.P., Vitaku E. (2021). Thermally conductive ultra-low-k dielectric layers based on two-dimensional covalent organic frameworks. Nat. Mater..

[2] Krishtab M., Stassen I., Stassin T., Cruz A.J., Okudur O.O., Armini S., Wilson C., De Gendt S., Ameloot R. (2019). Vapor-deposited zeolitic imidazolate frameworks as gap-filling ultra-low-k dielectrics. Nat. Commun..

[3] Zhang X., Zhang Y., Zhou Q., Zhang X., Guo S. (2019). Symmetrical “Sandwich” Polybutadiene Film with High-Frequency Low Dielectric Constants, Ultralow Dielectric Loss, and High Adhesive Strength. Ind. Eng. Chem. Res..

[4] Zhou D.L., Li J.H., Guo Q.Y., Lin X., Zhang Q., Chen F., Han D., Fu Q. (2021). Polyhedral Oligomeric Silsesquioxanes Based Ultralow-k Materials: The Effect of Cage Size. Adv. Funct. Mater..

[5] Li T.L., Hsu S.L.C. (2011). Preparation and Properties of Thermally Conductive Photosensitive Polyimide/Boron Nitride Nanocomposites. J. Appl. Polym. Sci..

[6] Guo Y.Q., Lyu Z.Y., Yang X.T., Lu Y.J., Ruan K.P., Wu Y.L., Kong J., Gu J.W. (2019). Enhanced thermal conductivities and decreased thermal resistances of functionalized boron nitride/polyimide composites. Compos. Part B-Eng..

[7] Mendes-Felipe C., Barbosa J.C., Goncalves S., Pereira N., Costa C.M., Vilas-Vilela J.L., Lanceros-Mendez S. (2020). High dielectric constant UV curable polyurethane acrylate/indium tin oxide composites for capacitive sensing. Compos. Sci. Technol..

[8] Ding C., Li R., Yu J., Wang X., Huang P. (2022). Synthesis of porous polyimide films with low dielectric constant and excellent mechanical properties by ambient pressure drying. J. Mater. Sci..

[9] Zhang Y., He J., Yang R. (2022). Ultra-low dielectric constant and high thermal stability of low-crosslinked polyimide with zinc tetraamino phthalocyanine. J. Mater. Sci..

[10] Mosadegh B., Xiong G.L., Dunham S., Min J.K. (2015). Current progress in 3D printing for cardiovascular tissue engineering. Biomed. Mater..

[11] Rengier F., Mehndiratta A., von Tengg-Kobligk H., Zechmann C.M., Unterhinninghofen R., Kauczor H.U., Giesel F.L. (2010). 3D printing based on imaging data: Review of medical applications. Int. J. Comput. Assist. Radiol. Surg..

[12] Lu B., Li D., Tian X. (2015). Development Trends in Additive Manufacturing and 3D Printing. Engineering.

[13] Kozior T., Kundera C. (2021). Viscoelastic Properties of Cell Structures Manufactured Using a Photo-Curable Additive Technology—PJM. Poymers.

[14] Bagheri A., Jin J.Y. (2019). Photopolymerization in 3D Printing. ACS Appl. Polym. Mater..

[15] Quan H., Zhang T., Xu H., Luo S., Nie J., Zhu X. (2020). Photo-curing 3D printing technique and its challenges. Bioact. Mater..

[16] Zhang J., Xiao P. (2018). 3D printing of photopolymers. Polym. Chem..

[17] Chen Y., Li J., Lu J., Ding M., Chen Y. (2022). Synthesis and properties of Poly(vinyl alcohol) hydrogels with high strength and toughness. Polym. Test..

[18] Wang J., Wang N., Xu D., Tang L., Sheng B. (2023). Flexible humidity sensors composed with electrodes of laser induced graphene and sputtered sensitive films derived from poly(ether-ether-ketone). Sens. Actuators B Chem..

[19] Liu J., Miao P., Zhang W., Song G., Feng J., Leng X., Li Y. (2022). Synthesis and characterization of interpenetrating polymer networks (IPNs) based on UV curable resin and blocked isocyanate/polyols. Polymer.

[20] Guo Y., Ji Z., Zhang Y., Wang X., Zhou F. (2017). Solvent-free and photocurable polyimide inks for 3D printing. J. Mater. Chem. A.

[21] Wei D., Liao B., Huang J., Zhang M., Pang H. (2019). Fabrication of castor oil-based hyperbranched urethane acrylate UV-curable coatings via thiol-ene click reactions. Prog. Org. Coat..

[22] Song Y., Wang J., Li G., Sun Q., Jian X., Teng J., Zhang H. (2008). Synthesis, characterization and optical properties of fluorinated poly(aryl ether)s containing phthalazinone moieties. Polymer.

[23] Rahmatabadi D., Aberoumand M., Soltanmohammadi K., Soleyman E., Ghasemi I., Baniassadi M., Abrinia K., Bodaghi M., Baghani M. (2023). 4D Printing-Encapsulated Polycaprolactone–Thermoplastic Polyurethane with High Shape Memory Performances. Adv. Eng. Mater..

[24] Aberoumand M., Soltanmohammadi K., Soleyman E., Rahmatabadi D., Ghasemi I., Baniassadi M., Abrinia K., Baghani M. (2022). A comprehensive experimental investigation on 4D printing of PET-G under bending. J. Mater. Res. Technol..

[25] Ge X., Yu L., Liu Z., Liu H., Chen Y., Chen L. (2019). Developing acrylated epoxidized soybean oil coating for improving moisture sensitivity and permeability of starch-based film. Int. J. Biol. Macromol..

[26] Lin Z., Ke Y., Peng X., Wu X., Zhang C., Zhao H., Feng P. (2022). Thermally Stable, Solvent Resistant, and Multifunctional Thermosetting Polymer Networks with High Mechanical Properties Prepared from Renewable Plant Phenols via Thiol–Ene Photo Click Chemistry. ACS Appl. Polym. Mater..

[27] Huang J., Yuan T., Ye X., Man L., Zhou C., Hu Y., Zhang C., Yang Z. (2018). Study on the UV curing behavior of tung oil: Mechanism, curing activity and film-forming property. Ind. Crop. Prod..

[28] Zhang F., Zong L., Bao F., Weng Z., Wang C., Wang J., Jian X. (2020). Novel phthalazinone-bearing tetrafunctional epoxy: Synthesis, characterization, and their toughening application for TGDDM system. Polym. Adv. Technol..

[29] Zhou Z.-X., Li Y.-W., Zheng Y.-Q., Luo Z., Gong C.-R., Xu Y., Wu L.-X. (2018). Synthesis and characterization of a dual-curing resin for three-dimensional printing. J. Mater. Sci..

[30] Yu Z.H., Cui A.Y., Zhao P.Z., Wei H.K., Hu F.Y. (2018). Preparation and properties studies of UV-curable silicone modified epoxy resin composite system. J. Appl. Biomater. Funct. Mater..

[31] Yang Z., Shan J., Huang Y., Dong X., Zheng W., Jin Y., Zhou W. (2020). Preparation and mechanism of free-radical/cationic hybrid photosensitive resin with high tensile strength for three-dimensional printing applications. J. Appl. Polym. Sci..

[32] Zhang X., Xu Y., Li L., Yan B., Bao J., Zhang A. (2019). Acrylate-based photosensitive resin for stereolithographic three-dimensional printing. J. Appl. Polym. Sci..

[33] Roig A., Ramis X., De la Flor S., Serra À. (2021). Sequential photo-thermal curing of (meth)acrylate-epoxy thiol formulations. Polymer.

[34] Chen Z., Zhao J., Yan S., Yuan Y., Liu S. (2015). Dielectric properties of photocrosslinkable polyimide/functional graphene oxide composites. Mater. Lett..

[35] Du N., Dal-Cin M.M., Robertson G.P., Guiver M.D. (2012). Decarboxylation-Induced Cross-Linking of Polymers of Intrinsic Microporosity (PIMs) for Membrane Gas Separation. Macromolecules.

[36] Qiu W., Chen C.-C., Xu L., Cui L., Paul D.R., Koros W.J. (2011). Sub-Tg Cross-Linking of a Polyimide Membrane for Enhanced CO_2_ Plasticization Resistance for Natural Gas Separation. Macromolecules.

[37] Kozior T., Mamun A., Trabelsi M., Sabantina L. (2022). Comparative Analysis of Polymer Composites Produced by FFF and PJM 3D Printing and Electrospinning Technologies for Possible Filter Applications. Ploymers.

