# Supplementary material for: Low Dielectric Constant Photocurable Fluorinated Poly (Phthalazinone Ether) Ink with Excellent Mechanical Properties and Heat Resistance
Source: Polymers (Basel). 2023 Mar 20;15(6):1531. doi: 10.3390/polym15061531 (PMC10051853; doi:10.3390/polym15061531)
Supplement: Supplementary file 1 [file polymers-15-01531-s001.zip › supplementary material.docx]

**n:m=3:2**

**Figure S1 The synthesis of FSt-FPPE.**

**Table S1 Characterization of molecular weights of FSt-FPPE.**

| **Name** | **RT(h)** | **Mn(g/mol)** | **PD** |
| --- | --- | --- | --- |
| **FSt-FPPE** | **14** | **6k** | **1.97** |

**
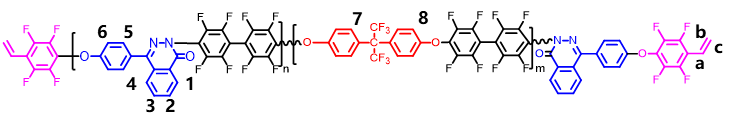
**

**
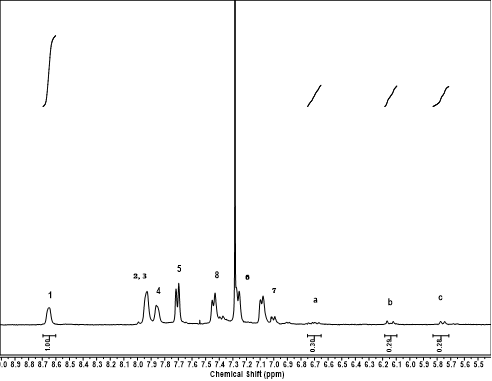
**

**Figure S2 1H NMR spectra of FSt-FPPE.**

**
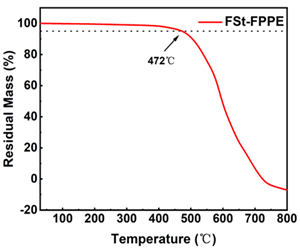
**
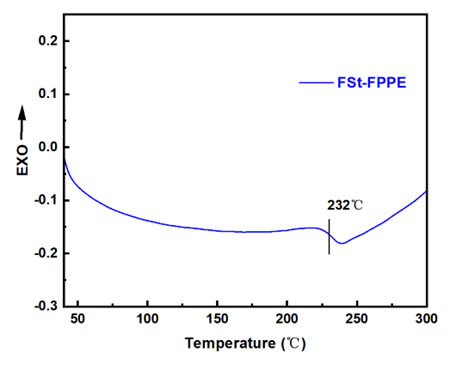


**Figure S3 TGA and DSC curves of FSt-FPPE.**

**DPGDA**

**TEOA**

**HPCK**

**Figure S4 The molecular structure for DPGDA, HPCK, and TEOA.**

**
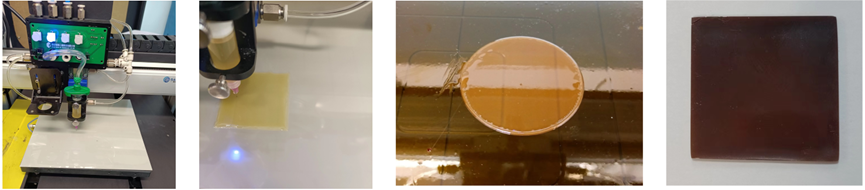
**

**Figure S5 Printing process and the image of the printed samples.**
